# Supplementary material for: Study on the influence of slope shape with numerical calculation models on slope safety during slope excavation
Source: PLoS One. 2023 Dec 14;18(12):e0292461. doi: 10.1371/journal.pone.0292461 (PMC10721094; doi:10.1371/journal.pone.0292461)
Supplement: Attachment — Submitted filename: PONE-D2311131-reviewer last.docx [file pone.0292461.s001.docx]

The work submitted to the PLOS ONE entitled as “Study on the influence of slope shape and geological structure on slope safety during slope excavation” is reviewed.

The proposed study examines the to understand the impact of slope shape, geological structure, and other conditions on slope stability of artificial slopes, calculation models for straight slope, concave slope, and convex slope are constructed based on the three slope shape characteristics. By changing the angles of upward and downward slope angles and analyzing the parameters of slope shape, joint spacing, and joint angle, discrete element software is used to calculate the slope safety factor. The reviewer believes this research paper could be interesting to the slope safety during slope excavation those who are interested in slope safety and slope shape models.

In general, a lot of calculation model has been carried out on this paper and the data is adequately analysed however it requires more technical contribution and improvements in the structural layout.

In general, the paper is well structured, and the data is well analysed and requires minor revision to be evaluated. I am suggesting the manuscript to be accepted for publication from the PLOS ONE; however, if the authors are willing to perform minor improvements/corrections on the submitted work.

Here are the minor improvements/corrections I suggest authors to review:

- Page 1. Heading should be changed. The reviewer recomment to change it as “Study on the influence of slope shape with numerical calculation models on slope safety during slope excavation”
- Page 1. Line 2, Instead of “good safety” try to use more technical and scientific terms.
- Page 1. Line 3-4, What does authors meant by “geological structures”. It should be removed from all sentences or it will be separately explained with another sub chapter.
- Page 2. Line 37 The geological structures should be briefly explain in another chapter. Most mainly sedimentary rocks should be given as an example .
- Page 3. Line 29, Page 4 Line 1 “The physical and mechanical parameters” is not well determined . Also rock mass materials are not sufficient in the table. The authors should add some more parameters (lithology, water condition, geological strength index etc. ) Try to use more wide used terminology as “ mechanical dynamic parameters” instead of “physical and mechanical parameters” or separate them with different tables.
- Page 4 . Check the table numbers. Last table should be Table 4 .
- Page 4,5,6 and 10. The headings of Chapter 3.1, 3.2, and 3.3 needs to be short.
- Page 14, 15 and 16. Change the format of captions for figures. It is different from the others. Check them all and use the template of the PONE journals.
- Page 16. Please indicate the recommendations and limitations of this study in the conclusion section

General comments

- The article requires English improvement.
- Resolution and size of the graphs should be improved for the final submission as they are too small to observe the reported behaviours.
- It is quite clear that there is a relationship between the slope angle and slope factor with mechanical parameters such as cohesion and friction angle. Authors can highlight this relationship by using simple statistical tools and developed simple regression models so one can predict one another by simply testing one specimen at certain density. Such approach will increase the novelty of this paper.
