# Supplementary material for: Study on the influence of slope shape with numerical calculation models on slope safety during slope excavation
Source: PLoS One. 2023 Dec 14;18(12):e0292461. doi: 10.1371/journal.pone.0292461 (PMC10721094; doi:10.1371/journal.pone.0292461)
Supplement: Attachment — Submitted filename: Response to Reviewers.docx [file pone.0292461.s002.docx]

Response to Reviewers

1. Page 1. Heading should be changed. The reviewer recomment to change it as “Study on the influence of slope shape with numerical calculation models on slope safety during slope excavation”

**Response**: Agreed, it has been modified according to the opinions.

2. Page 1. Line 2, Instead of “good safety” try to use more technical and scientific terms.

**Response**: Agreed, it has been modified according to the opinions.

3. Page 1. Line 3-4, What does authors meant by “geological structures”. It should be removed from all sentences or it will be separately explained with another sub chapter.

**Response**: Agreed, the 'geological structures' in the article has been changed to 'geological setting'.

4. Page 2. Line 37 The geological structures should be briefly explain in another chapter. Most mainly sedimentary rocks should be given as an example .

**Response**: Agreed, the 'geological structures' in the article has been changed to 'geological setting'.

5. Page 3. Line 29, Page 4 Line 1 “The physical and mechanical parameters” is not well determined . Also rock mass materials are not sufficient in the table. The authors should add some more parameters (lithology, water condition, geological strength index etc. ) Try to use more wide used terminology as “ mechanical dynamic parameters” instead of “physical and mechanical parameters” or separate them with different tables.

**Response**: Agreed, relevant parameters have been added to the article.

6. Page 4 . Check the table numbers. Last table should be Table 4 .

**Response**: Agreed, it has been modified according to the opinions.

7. Page 4,5,6 and 10. The headings of Chapter 3.1, 3.2, and 3.3 needs to be short.

**Response**: Agreed, it has been modified according to the opinions.

8. Page 14, 15 and 16. Change the format of captions for figures. It is different from the others. Check them all and use the template of the PONE journals.

**Response**: Agreed, it has been modified according to the opinions.

9. Page 16. Please indicate the recommendations and limitations of this study in the conclusion section

**Response**: Agreed, this part has been supplemented in the conclusion of the article.

**General comments**

1. The article requires English improvement.

**Response**: The article has been polished and modified for language issues.

2. Resolution and size of the graphs should be improved for the final submission as they are too small to observe the reported behaviours.

**Response**: The unclear images in the article have been replaced. The images in the article have been uploaded as attachments.

3. It is quite clear that there is a relationship between the slope angle and slope factor with mechanical parameters such as cohesion and friction angle. Authors can highlight this relationship by using simple statistical tools and developed simple regression models so one can predict one another by simply testing one specimen at certain density. Such approach will increase the novelty of this paper.

**Response**: Agreed. Analysis of the calculated data was conducted based on the suggestions of the reviewers, but due to the large number of abrupt changes in the trend of data changes, it is necessary to conduct in-depth analysis and research to establish the trend relationship of data changes using regression models. Therefore, the review comments will be part of the subsequent research.
